# Roadmap to Wellness: Exploring Live Customized Music at the Bedside for Hospitalized Children

**DOI:** 10.3389/fonc.2018.00021

**Published:** 2018-02-07

**Authors:** R. Serene Perkins, Maura Boyce, Megan C. Byrtek, Regina C. Ellis, Cindy Hill, Paul S. Fitzpatrick, Shaban Demirel

**Affiliations:** ^1^Legacy Research Institute, Legacy Health, Portland, OR, United States; ^2^Children’s Cancer Association, Portland, OR, United States; ^3^Randall Children’s Hospital at Legacy Emanuel, Legacy Health, Portland, OR, United States

**Keywords:** music, live, customized, pediatrics, postoperative, neuropsychology, transformative, joy

## Abstract

**Background:**

Randomized trials on clinical outcomes of music are conflicting, with few performed in the postoperative pediatric population. We aimed to determine if there was a benefit of a live, customized bedside music delivery program (MyMusicRx^®^) for children hospitalized after pediatric surgery. We present our perspective on the utility of music medicine, review others’ work in this area, and discuss future directions.

**Methods:**

All admitted postsurgical patients aged between 5 and 18 years were considered. One live, customized music session was delivered by a MyMusicRx^®^ music specialist to intervention participants, and compared with matched controls who did not receive music intervention. Pain, cumulative analgesia dosage, and vital signs within 12 h after unit arrival were compared between groups.

**Results:**

Thirty-two participants (16 intervention, 16 controls; 8:8 females:males per group) were enrolled. No differences in age, surgery length, or duration of music intervention were found between groups. No differences in pain scores (*p* = 0.73), heart rate (*p* = 0.82), respirations (*p* = 84), narcotic (*p* = 0.92) or non-narcotic medication usage (*p* = 0.88, 0.86, 0.95; ibuprofen, acetaminophen, and ketorolac, respectively), or time to first narcotic dose (*p* = 0.64) were found.

**Conclusion:**

A single music intervention in the acute postoperative period did not appear to be adequate to augment traditional methods of pain and hemodynamic control. Prior studies have similar outcome measures but conflicting results. We did not evaluate psychological well-being, patient engagement, or family perception in this pilot study. Future directions include developing and validating a tool that explores the observable impact of music medicine on children’s emotions and behaviors.

## Music Medicine: Two Decades of Experience in Hospital Settings

In 2015, the Children’s Cancer Association (CCA), in partnership with Randall Children’s Hospital (RCH, Portland, OR, USA) and the Legacy Research Institute (Portland, OR, USA), aimed to determine if there was a measureable benefit to a well-established music delivery program for hospitalized children. Based upon over two decades of experience bringing live, customized music to the bedside and hundreds of patient testimonials, CCA leaders hoped that their methods could be shown to mitigate stress, anxiety, and perception of pain. These methods include trained music specialists utilizing music carts filled with instruments for teaching and exploration, as well as iPads with musical apps and online content including artist greetings, concerts, and music lessons. The comprehensive program was coined MyMusicRx^®^ by the organization’s founder, Regina Ellis, whose daughter Alexandra died of a childhood cancer.

Since 1995, CCA has leveraged music, friendship, nature, and resources to create transformative moments of joy for families facing cancer and other serious illnesses, with a vision to make MyMusicRx^®^ best practice in pediatric hospitals nationally. Due to growth of its programs over the years, CCA now delivers music medicine to all pediatric patients, regardless of diagnosis; as the model of joy should not be limited to cancer patients. MyMusicRx^®^ provides live, customized music at bedsides from the emergency room to the chemotherapy unit. Customization of the music experience by MyMusicRx^®^ specialists is based on patient age, interest, and other intangible factors. These tailored interactions range in music appropriate for the situation (i.e., mellow if a child is aiming to rest or fall asleep, engaging to distract the child during a procedure, participatory if the child is able to interact) and can include family members or care providers.

The usefulness of non-pharmacological interventions in health-care settings has been studied extensively. Many hospitals utilize integrative therapies (1–4) to relieve pain and anxiety, and to bring joy in a difficult setting. While its use in health-care settings is not new, music is making its way into operating and recovery rooms in an effort to reduce postoperative discomfort. In the largest systematic review to date of music and postoperative recovery, music improved outcomes and was significantly associated with reduced pain, anxiety, and analgesia usage, as well as increased patient satisfaction (5). In this review of 76 eligible randomized controlled trials, benefits remained even when music was delivered during general anesthesia.

Most publications regarding the use of music in the postoperative environment involve adult patients. Given the greater difficulty in assessing and managing pain in children, as well as their greater likelihood of being anxious in unfamiliar settings, we aimed to explore the impact of music medicine in pediatric surgical patients. Leveraging the collaboration between CCA and RCH, we studied the effects of live, customized music in the pediatric surgical population. At the time of protocol development, music intervention had not been examined in the pediatric postoperative setting; furthermore, the concept of creating transformative moments of joy had not been investigated using scientific methodologies.

## Pilot Study Design

All postsurgical patients between age 5 and 18 years admitted to RCH acute inpatient units and not intubated were considered. Because surgical patients with cancer are lower in frequency (approximately 3% of total surgeries/year), we chose to include patients with all surgical diagnoses, who could be matched to a control population, and who represented a cross-section of those who might otherwise receive MyMusicRx^®^ services. Intervention patients received at least 5 min of live, customized music at their bedside, delivered by a MyMusicRx^®^ specialist, within 4 h of arrival to the unit. The control group was selected from patients that did not receive the intervention and matched to the intervention group on a case-by-case basis using age (±1 year), gender, and type of surgery. Groups were compared based on nurse-assessed or self reported (depending on patient age) postoperative pain (0–10 scale), cumulative dose of analgesia, and vital signs (blood pressure, heart rate, and respiratory rate) within the 12-h period after arrival to the unit and after the intervention ended. Information gathered was part of standard clinical assessments; no study-specific measurements were made, and no hospital protocols were altered. The study was approved by the Legacy Health Institutional Review Board; a waiver of informed consent was granted due to the study’s low risk nature, and the fact that study foreknowledge could have altered patients’, parents’, and caregivers’ reports and perceptions of pain. All patients whose data were used received an information sheet after study completion.

The primary outcome measure (pain on 0–10 scale) was non-continuous; however, it was nearly normal within its range. Consequently, a generalized linear model was constructed that accounted for the fact that several pain measures were made per child during the postoperative period (clustering of pain within child). Generalized linear models were used for many of the secondary outcome measures that were continuous and near-normally distributed (narcotic and non-narcotic drug usage). For comparisons of continuous descriptive measures (age, length of surgery, etc.), the paired *t*-test was used to seek between group differences.

## Results: The Perspective on Music as Medicine

Sixteen participants in the intervention group were matched to 16 controls (8 males, 8 females per group). There was no significant difference in average age between groups (12.23; range 7.3–17.47 versus 12.26; 7.56–17.03 years, respectively; *p* = 0.98). Length of surgery was not significantly different between groups (1:46; range 0:29–4:42 versus 2:00; 0:59–6:39 hours, respectively; *p* = 0.61). In the intervention group, the average duration of the music intervention was 16.6 (range 5–27) minutes. There were no significant differences between groups in pain scores (*p* = 0.73), heart rate (*p* = 0.82), respirations (*p* = 84), narcotic (morphine equivalent/kg; *p* = 0.92), or non-narcotic medication usage (*p* = 0.88, 0.86, 0.95, respectively, for ibuprofen, acetaminophen, and ketorolac), or time to first narcotic dose (*p* = 0.64). The use of postoperative anxiolytics was found to be negligible and did not warrant comparison.

Therefore, in the immediate 12-h postoperative period, standard measures of pain (nursing pain assessment, self report, and pain medication use) and hemodynamics (vital signs) were equivalent between groups in this exploratory pilot study. A single music intervention in the acute postoperative period does not appear to be enough to affect pain scores, vital signs, and medication usage. While one could conclude that tailored music delivery does not improve pain or hemodynamics in this setting, one could also argue that a single music intervention of short duration is not adequate to augment traditional methods of achieving pain and hemodynamic control. Furthermore, the framework of this study was not designed to mirror CCA’s MyMusicRx^®^ bedside interactions, which are focused on transforming the moment for patients, but instead were bold and ambitious in exploring the lasting effects of a single music intervention on postoperative pain and anxiety.

## Music Medicine: Year in Review and Neuropsychology as a Common Thread

Our study addresses a critical gap in pediatric oncology research: the role of integrative therapies to combat the adverse effects of stress in cancer (6). Several randomized controlled trials examining the clinical outcomes of music intervention in surgical patients show conflicting results. We reviewed 76 randomized clinical trials between November 2015 and January 2017 involving music intervention in surgical patients; only three were in the pediatric population. These studies tended to focus on music therapy as an intervention—a distinctly different approach to music delivery compared with MyMusicRx^®^. The American Music Therapy Association defines music therapy as “music interventions to accomplish individualized goals within a therapeutic relationship” (7). Music therapy in these studies tended to involve complementary techniques such as hypnosis, guided imagery, controlled beat or tempo, and relaxation, with multiple or lengthy sessions employed to achieve a desired effect, such as reduction in heart rate (8, 9). These studies were often limited by inconsistent methodologic quality and insufficient details, or investigator and subject bias. Furthermore, many results were conflicting, with some showing significant improvements in standard measures of pain and anxiety and others demonstrating none or contradictory findings.

For example, in a study of 60 patients undergoing surgery for lung carcinomas, music therapy showed significant improvements in both pain and anxiety scores, as well as decreased vital signs and patient-controlled narcotic analgesia requirements. Subjects received one 15-min intervention using intentional muscle relaxation with light and moderate hypnosis, language, and relaxing music preoperatively, followed by four additional interventions postoperatively in 1-h phases. It was difficult to determine whether music, hypnosis, time, and number of treatments, or a combination led to this positive effect (9).

In a similar study, thoracic surgery patients receiving music therapy with recorded “soft,” “melodious,” and “pleasant rhythms,” at 60–80 beats/min or less, versus no music therapy, had improved pain and anxiety scores and decreased systolic blood pressure. After eliminating distractions, intervention patients were guided by the researcher to keep their breathing smooth, relaxed, and focused on the music. However, patient-controlled analgesia requirements were equivalent in this study. Again, it is difficult to determine whether it was the music therapy itself, or incorporation of mind–body interventions, such as guided hypnosis, that led to the positive effect (8).

While the two aforementioned studies incorporated music selected by the investigator or administrator, others used patient-selected or patient-preferred music. For example, in a study of live versus recorded music in ambulatory breast surgery patients, Global Anxiety Visual Analog Scale scores significantly improved from baseline when any form of music was offered. Participants were asked to report the song that most reduced their anxiety; this song was subsequently used for the intervention. While anxiety scores and time to discharge readiness improved, both selection and interviewer bias were introduced. Anesthetic requirements were equivalent in this study (10). Similarly, a large (*n* = 400) cohort of patients undergoing two sessions of shockwave lithotripsy and receiving recorded, self-selected music at different time intervals showed improved anxiety and pain scores, but equivalent vital signs measurements. These patients were not masked from the time at which the intervention was received, and therefore the study was subject to high potential bias (11).

Another study recruited a cohort of transplant recipients and those on the wait list—both groups considered to be under high psychosocial stress. “Coping-infused dialog,” where patients identified stressors and received relationship support, in addition to “live, familiar music” in structured, predictable, and sequential sessions, were administered to the transplant recipients; wait listed patients were used as the control group. The results were conflicting, with global mood scale improving in the intervention group and pain scores remaining equivalent. Again, it is difficult to determine whether the music itself, structure and predictability, autonomy in music selection, coping-infused dialog, or a combination of these led to the improvements in affect. Although unknown, it is conceivable that the receipt of a transplant itself may have played some role in improving affect (12). It is also possible that behavioral conditions, such as choice of music and anticipation of stress reduction, may have played a role in both studies.

Two systematic reviews on music in the postoperative setting presented conclusions consistent with our findings. Hole et al. raised several concerns around the quality of study design. Where masking was not done or clarified, heterogeneity was high; even increased pain, anxiety, and analgesic use was found when patients were conscious (5). McConnell et al. also highlighted that combining the use of coping techniques, environmental adjustments, and guided imagery purported a high risk of bias in determining whether improved pain scores were due to music or to other interventions (13). Attention should be given to a recent systematic review performed in the pediatric surgical population, where only three randomized controlled trials were eligible for analysis of the effects of music—either live or recorded, used as entertainment or to achieve a therapeutic goal (14). Of these three trials, conflicting effects on pain and anxiety were reported, making it difficult for the authors to draw strong conclusions.

Finally, in a paper commissioned by the Musical Connections Program of Carnegie Hall’s Weill Music Institute, the authors note: “In the field of music and health … research has focused away from the global (is music good for patients’ feeling of well being?) and toward the highly particular (can a hypothesized stress reduction outcome be proven in a statistically valid way for oncology patients before chemotherapy?).” The authors highlight challenges of study design and statistical validity that make it difficult to draw valid conclusions about the efficacy of music to influencing measurable health-related outcomes. Furthermore, research directions in neuroscience and neuropsychology have also “posed challenges for those advocating for music therapy as the ‘evidence’ often does not hold up to the highest forms of scientific scrutiny” (15).

The inconclusive results in the existing literature led us to question whether standard measures of pain and anxiety (subjective scoring systems and hemodynamic parameters) were too narrow, or did not capture the effects of music on patients. Furthermore, our study did not include patient or family feedback or measure joy, smiles, laughter, and other mood indicators not typically assessed by standardized methods. Our review led us to a subtle, yet profound common thread: repeated, consistent “doses” of music—as opposed to a single, brief interaction—appear to have a measurable effect during illness. This raises the question as to whether patients with chronic illnesses such as cancer could benefit from more sustained, stress-mitigating interventions over time. Our hospital’s child life program includes support elements to distract and normalize the environment (art therapy, dress-up, pet therapy, treasure chests, children’s garden, etc.), and such interventions have proven efficacy (16, 17). Thus, combining music with other patient-centered interventions may increase its effectiveness. According to Collins et al., “research requires standardized interventions while music therapy likely requires interventions tailored to the individual” (18); this is what our study aimed to accomplish. Our pilot study was designed to explore the short-term effects of one short music interaction on physiologic parameters, standardized pain scores, and the amount of medication given in the immediate postoperative period, and not how MyMusicRx^®^ “transforms the moment” for children and their families on a daily basis. We believe that several transformative moments have an additive effect on a child’s well-being. This is true for other stress-mitigating strategies in chronically ill children; cognitive-behavioral interventions in asthma (19, 20), psychosocial family systems (21) in chronic illness, and patient empowerment through therapeutic video games in pediatric cancer (22). Regardless of the modality, stress management using cognitive methods is promising and requires further study.

## The Impact of Music Medicine: Creating Transformative Moments of Joy

To determine how to best to capture the impact of music for hospitalized children, we reflected on our experience providing music in the pediatric hospital setting. Feedback from families and our music specialists conveyed similar and identifiable themes: laughter, relaxation, smiles, and distraction. We aimed to create an objective methodology to capture these emotive and behavioral indicators, to better understand and measure the impact of music in the moment. In addition, since our program allows children to tailor their experience, we did not focus on a single “right” outcome, but a suite of key outcomes that capture a child’s experience. Our initial criteria were to design a tool that was concise, objective, and appropriate for interactions lasting an average of 20 min. While we recognize the potential bias in having a music specialist provide the intervention and record the response, we do not want to influence a child’s experience by inserting an additional observer into the hospital room. Given the sensitivity of the medical environment and the length of each interaction, a tool that could be used by the MyMusicRx^®^ specialist during the interaction is most desirable.

We first searched existing validated tools appropriate to our program’s mission and found one that provided a framework on which to build our instrument. The Children’s Emotional Manifestation Scale (CEMS) provides an “objective and operationalized behavioral scale … to document children’s emotional responses during stressful medical procedures” (22). Since the CEMS was designed to capture emotions during a stressful medical procedure, it only reflects one side of the emotional spectrum (agitated to calm). We aimed for our instrument to also document a positive emotional response to an intervention, such as smiling, laughing, or becoming relaxed. We chose to use the CEMS as a template, and modify it to capture the fuller range of emotions that our program seeks to affect. There continue to be limitations in measuring the full range of a youth’s emotional reaction in response to an intervention such as music in the medical setting. The CEMS was developed because of this gap; we agree and hope to provide an expanded scale that demonstrates the positive impact of music and is valuable in determining ways to mitigate stress in the hospital setting.

We also reviewed 69 pieces of written feedback obtained from MyMusicRx^®^ served families to identify key words describing the emotional or behavioral changes music had on a child. Themes become apparent in the language used, and its alignment with the intended program outcomes. This included smiles seen for the first time since hospitalization, visible relaxation of the face and body, a happy change in attitude, and distraction during procedures. We used this feedback to develop a set of indicators with rating anchors, a quantitatively measurable, observational scale to describe a child’s response to music. Testing of an outcomes matrix to measure and analyze indicators of emotion and self expression is in progress.

In conclusion, this pilot study on the impact of a single music intervention was somewhat inconclusive. While a single music intervention of short duration does not appear to be adequate to augment traditional methods of achieving pain and hemodynamic control, the outcome measures were limited, as further demonstrated by inconsistent results in the literature. Moreover, research from neuroscience and neuropsychology disciplines provides us with a compass to redirect investigations of the impact of music medicine. Developing and validating a tool that explores how music medicine affects children’s emotions and behaviors may help us to demonstrate an observable impact of music medicine. This new research direction may provide new clinical practices for music medicine interactions in conjunction with traditional interventions in pediatric hospitals.

## Ethics Statement

The study was approved by the Legacy Health Institutional Review Board; a waiver of informed consent was granted due to the low risk nature of the study, and the fact that foreknowledge of the study could have altered patients’, parents’, and caregivers’ reports and perceptions of pain (the primary outcome measure).

## Author Contributions

RP made substantial contributions to the design and interpretation of data for the work, drafting and revising it critically for important intellectual content, final approval of the version to be published, and agreed to be accountable for all aspects of the work in ensuring that questions related to the accuracy or integrity of any part of the work are appropriately investigated and resolved. MB contributed to the interpretation of data for the work, drafting and revising it critically for important intellectual content, final approval of the version to be published, and agreed to be accountable for all aspects of the work in ensuring that questions related to the accuracy or integrity of any part of the work are appropriately investigated and resolved. MCB and RE made substantial contributions to the conception and design of the work, revising it critically for important intellectual content, final approval of the version to be published, and agreed to be accountable for all aspects of the work in ensuring that questions related to the accuracy or integrity of any part of the work are appropriately investigated and resolved. CH made substantial contributions to the design of the work, revising it critically for important intellectual content, final approval of the version to be published, and agreed to be accountable for all aspects of the work in ensuring that questions related to the accuracy or integrity of any part of the work are appropriately investigated and resolved. PF made substantial contributions to the acquisition of data for the work, drafting it for important intellectual content, final approval of the version to be published, and agreed to be accountable for all aspects of the work in ensuring that questions related to the accuracy or integrity of any part of the work are appropriately investigated and resolved. SD made substantial contributions to the design of the work, the analysis and interpretation of data for the work, revising it critically for important intellectual content, final approval of the version to be published, and agreed to be accountable for all aspects of the work in ensuring that questions related to the accuracy or integrity of any part of the work are appropriately investigated and resolved.

## Conflict of Interest Statement

The funding for the costs of conducting the research was provided by Randall Children’s Hospital and the Children’s Cancer Association (CCA) as joint sponsors of the research. CCA does hold a registered trademark for MyMusicRx^®^, the name of the service under research for this project. The PI, RP, is a Legacy Health employee and member of the Board of Directors of CCA but was not paid additional compensation as a result of CCA funding. Although existing Legacy Research Institute staff were assigned to conduct the research, none of them were paid additional compensation as a result of CCA funding. None of the Legacy Research Institute study staff hold patents or financial interests in CCA trademarks. MB, MCB, and RE are employees of CCA and the cosponsor of the research.

## Abbreviations

CCA, Children’s Cancer Association; CEMS, Children’s Emotional Manifestation Scale; RCH, Randall Children’s Hospital.
